# A rare case of superior mesenteric venous and portal vein thrombosis in complicated appendicitis

**DOI:** 10.1093/jscr/rjae580

**Published:** 2024-09-11

**Authors:** John Paul Bustamante, Claire Caplan, Joanna Sajdlowska, Zamaan Hooda, Melissa Warta

**Affiliations:** Department of Surgery, St Joseph’s University Medical Center, 703 Main Street, Paterson, NJ 07503, United States; Department of Surgery, St Joseph’s University Medical Center, 703 Main Street, Paterson, NJ 07503, United States; Department of Surgery, St Joseph’s University Medical Center, 703 Main Street, Paterson, NJ 07503, United States; Department of Surgery, St Joseph’s University Medical Center, 703 Main Street, Paterson, NJ 07503, United States; Department of Surgery, St Joseph’s University Medical Center, 703 Main Street, Paterson, NJ 07503, United States

**Keywords:** portal vein thrombosis, superior mesenteric vein thrombosis, acute appendicitis

## Abstract

Superior mesenteric venous (SMV) thrombosis is a rare complication of severe appendicitis. Early recognition is due to improved imaging modalities, which ultimately lead to more prompt intervention. Despite being an uncommon phenomenon, SMV thrombosis can have complications stemming from venous hypertension, such as gastric and esophageal varices, bowel ischemia, sepsis, and death. As this is a rare phenomenon, specific treatment guidelines and algorithms are lacking in the current literature. This case report describes a 23-year-old male patient whose recovery from a laparoscopic appendectomy was complicated with both an SMV and portal vein thrombosis.

## Introduction

Superior mesenteric venous (SMV) thrombus is a rare complication of severe appendicitis [1]. Early recognition and improved imaging and guidelines have led to more prompt intervention [1].

Suppurative pylephlebitis is thrombophlebitis that affects the portal venous system, and the typical culprit is an infectious etiology from an intra-abdominal or pelvic organ. With appendiceal and colonic sources, the infection is spread via the venous drainage of the system, which ultimately leads to suppurative pylephlebitis. In the case of hepatobiliary infections, suppurative pylephlebitis occurs secondary to the contiguous nature of the involved organs, which is seen in cases of cholecystitis, pancreatitis, and infectious hepatitis. The overall incidence of suppurative pylephlebitis is rare, reportedly <2.7 per 100 000 people per year [2]. As this disease process is not often encountered by clinicians, there are not clear management guidelines available that we were able to locate.

In this case report, we discuss this rare entity occurring in an otherwise healthy 23-year-old male patient following the diagnosis of acute appendicitis treated by laparoscopic appendectomy.

## Case report

A 23-year-old male patient without significant past medical or surgical history presented to the emergency department complaining of 3 days of abdominal pain, fevers, nausea, and emesis. Upon presentation, the patient was afebrile and slightly tachycardic with an otherwise unremarkable hemodynamic status. His physical examination revealed lower abdominal tenderness without peritoneal signs, and his laboratory studies did not show any significant abnormalities. Computed tomography (CT) of the abdomen and pelvis was obtained for further evaluation, which demonstrated a 12 mm appendix with prominent fat stranding without any evidence of perforation.

This finding of a dilated appendix, in combination with abdominal tenderness and complaints of subjective fever, raised suspicion for acute appendicitis. The patient was started on intravenous antibiotics and was urgently taken to the operating room for surgical intervention. Ultimately, the patient underwent an uncomplicated laparoscopic appendectomy. Intra-operatively, it was noted that the appendix did appear very inflamed and was adhered to the cecum. However, there was no evidence of perforation or abscess formation. The appendix was sent as a specimen to pathology for further analysis, which later revealed severe appendicitis and peri-appendicitis. Postoperatively, the patient demonstrated improvement of symptoms and was ultimately discharged the same day as the operation.

**Figure 1:**
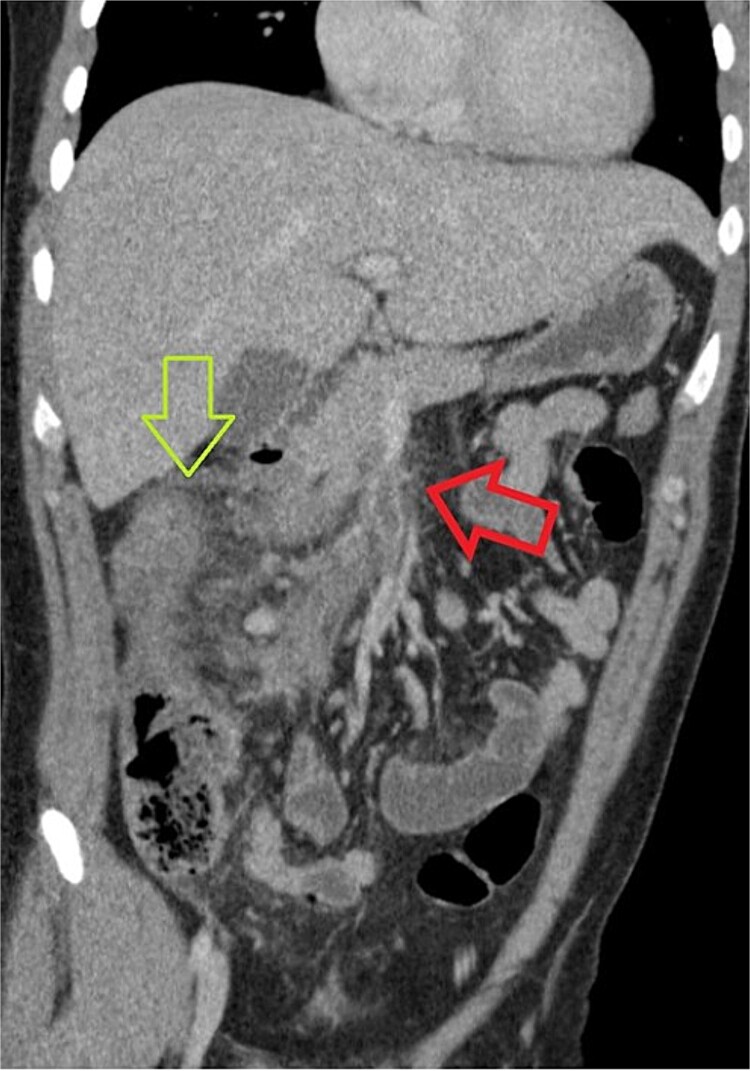
CT of abdomen and pelvis showing a thrombus within the superior mesenteric vein, identified with a red (or horizontal) arrow. Some linear foci which may be branching of hypoattenuation in segment 8 of the liver which possibly related to distal branches of the right portal vein and could represent early portal vein thrombosis. The green (or vertical) arrow identifies colonic edema at the hepatic flexure.

One week later, the patient returned to the emergency department with complaints of severe generalized abdominal pain for several days, fever, and emesis. During this presentation, the patient was noted to be febrile to 38°C and tachycardic. Laboratory studies were obtained and complete blood count was remarkable for a white blood cell count of 22.7 with a left shift. However, lactic acid levels were within normal limits. Upon performing a physical examination, there was diffuse and moderate tenderness to the right side of the abdomen and epigastrium. Given the recent surgery, there was concern for a potential complication. His presentation prompted another CT of the abdomen and pelvis, and this showed a superior mesenteric vein thrombosis with a discontinuous right portal vein thrombosis near the dome of the diaphragm. Additionally, there was notable associated edema of the ascending and transverse colon suggesting colitis secondary to poor venous drainage (Fig. 1). Due to the finding of thromboses, a D-dimer level was obtained and found to be 3.7.

Given these CT findings, surgery was consulted for further evaluation. His initial treatment began with administration of intravenous Zosyn following the obtainment of blood cultures. Ultimately these blood cultures were negative. Importantly, systemic anticoagulation with a heparin infusion was immediately initiated. The hematology service was also consulted, and based on their evaluation, no further hematological diagnostic testing was necessary as the thromboses were provoked from the acute appendicitis. Due to the requirement of systemic anticoagulation, evidence of infection and concern for potential sepsis, the patient was initially under the management of the surgical intensive care unit. As his heparin drip reached therapeutic levels, his abdominal pain began to subside and his vital signs returned to normal limits over the next 2 days. The patient was also able to tolerate a regular diet and demonstrated adequate functional performance of daily activities. On hospital Day 3, he was transitioned from heparin infusion to oral apixaban with plan to continue this anticoagulation regimen for the next 6 months. On hospital Day 5, the patient was transitioned from IV Zosyn to oral Augmentin and discharged with a 10 day course. He returned to the outpatient Surgery clinic 2 weeks after discharge and again reported a resolution of symptoms and good adherence. As he was no longer experiencing symptoms, no further imaging was obtained. Subsequently, he was lost to follow up with the Hematology service.

## Discussion

Pylephlebitis is a rare but morbid complication of intra-abdominal infection that was first described in the early 1800s. Initially, it was thought to be most associated with appendicitis, though recent literature suggests that diverticulitis is the most common underlying cause [3]. Due to the rarity of this disease process, there are varying recommendations and guidelines for treatment. Management often relies on broad-spectrum intravenous antibiotics, with or without anticoagulation. In a review that included cases of pylephlebitis diagnosed from 2010 to 2021, 94.2% of the patients received intravenous antibiotics, and 76.7% of patients received systemic anticoagulation with intravenous heparin [4].

Regardless of the presence of bacteremia, the current literature suggests the use of empiric antibiotics [4, 5]. However, the use of anticoagulation remains a controversial aspect of treatment. Proponents of using systemic anticoagulation believe that its use can potentially avoid further complications that are related to clot burden, including bowel ischemia, portal hypertension, and clot propagation [5]. Despite the mixed consensus, there has never been a randomized trial to further elucidate the role of anticoagulation in pylephlebitis.

Ultimately, our case highlights a rare complication of acute appendicitis in an otherwise healthy young adult male patient. Pylephlebitis management lacks agreed upon management due to a paucity of updated and targeted research, and this matter warrants further investigation.

## Conflict of interest statement

None declared.

## Funding

None declared.
